# Psychometric properties of leadership scales for health professionals: a systematic review

**DOI:** 10.1186/s13012-021-01141-z

**Published:** 2021-08-28

**Authors:** Melissa A. Carlson, Sarah Morris, Fiona Day, Ann Dadich, Annika Ryan, Elizabeth A. Fradgley, Christine Paul

**Affiliations:** 1 Hunter Cancer Research Alliance, Newcastle, New South Wales Australia; 2 School of Medicine and Public Health, University of Newcastle, Callaghan, New South Wales Australia; 3 Calvary Mater Newcastle, Waratah, New South Wales Australia; 4 Centre for Oncology Education and Research Translation (CONCERT), Western Sydney University, Penrith, Australia

**Keywords:** Leadership, Change champions, Psychometrics, Implementation, Healthcare

## Abstract

**Background:**

The important role of leaders in the translation of health research is acknowledged in the implementation science literature. However, the accurate measurement of leadership traits and behaviours in health professionals has not been directly addressed. This review aimed to identify whether scales which measure leadership traits and behaviours have been found to be reliable and valid for use with health professionals.

**Methods:**

A systematic review was conducted. MEDLINE, EMBASE, PsycINFO, Cochrane, CINAHL, Scopus, ABI/INFORMIT and Business Source Ultimate were searched to identify publications which reported original research testing the reliability, validity or acceptability of a leadership-related scale with health professionals.

**Results:**

Of 2814 records, a total of 39 studies met the inclusion criteria, from which 33 scales were identified as having undergone some form of psychometric testing with health professionals. The most commonly used was the Implementation Leadership Scale (*n =* 5) and the Multifactor Leadership Questionnaire (*n* = 3). Of the 33 scales, the majority of scales were validated in English speaking countries including the USA (*n* = 15) and Canada (*n* = 4), but also with some translations and use in Europe and Asia, predominantly with samples of nurses (*n* = 27) or allied health professionals (*n* = 10). Only two validation studies included physicians. Content validity and internal consistency were evident for most scales (*n* = 30 and 29, respectively). Only 20 of the 33 scales were found to satisfy the acceptable thresholds for good construct validity. Very limited testing occurred in relation to test-re-test reliability, responsiveness, acceptability, cross-cultural revalidation, convergent validity, discriminant validity and criterion validity.

**Conclusions:**

Seven scales may be sufficiently sound to be used with professionals, primarily with nurses. There is an absence of validation of leadership scales with regard to physicians. Given that physicians, along with nurses and allied health professionals have a leadership role in driving the implementation of evidence-based healthcare, this constitutes a clear gap in the psychometric testing of leadership scales for use in healthcare implementation research and practice.

**Trial registration:**

This review follows the Preferred Reporting Items for Systematic Reviews and Meta-Analyses (PRISMA) (see Additional File 1) (PLoS Medicine. 6:e1000097, 2009) and the associated protocol has been registered with the PROSPERO International Prospective Register of Systematic Reviews (Registration Number CRD42019121544).

**Supplementary Information:**

The online version contains supplementary material available at 10.1186/s13012-021-01141-z.

Contribution to the literature

- Little is known about how to identify and measure leadership traits and behaviours in health professionals despite the importance of clinical leadership for achieving practice change.
- The review identified a small number of scales (*n* = 7) which may be sufficiently sound to be used with nurses and allied health professionals.
- Although two studies included physicians, no scales were identified as providing sound assessment of physicians’ leadership traits and behaviours.
- There is an opportunity to advance the science of implementation through further validation of existing scales with physicians and males, and in assessing and understanding gender and cultural differences in implementation leadership.

## Introduction

### Background

#### The challenge of improving research translation or implementation

Translation of scientific knowledge to routine, evidence-based practice in healthcare settings ensures optimal care and improved outcomes for patients [1, 2]. Despite this, the translation of research knowledge to evidence-based practice is often slow or poor [3–6]. A foundational study by McGlynn [7, 8] found that during a two-year period between 1998 and 2000, patients in the United States receive 55% of evidence-based care with great variance in the rate of evidence-based care received among medical conditions. Furthermore, a 2005 systematic review by Schuster et al. [9] found 30–40% of patients were missing out on treatment that has been proven to be effective, while 20–25% of patients were receiving treatments that they do not need or that can cause them harm. A more recent Australian study by Runciman et al. [10] in 2012 with a sample of 1154 participants found that participants received appropriate care at 57% of healthcare encounters, again varying across medical conditions (from 32 to 86%). McGlynn [8] suggests that despite attempts to address these deficits in evidence-based care, there have been no large-scale studies in the United States measuring the provision of evidence-based care since 2003 and that although smaller studies indicate there have been improvements in some areas, there has been little change in healthcare overall. This failure to translate knowledge to evidence-based practice can result in poor outcomes for patients including sub-optimal treatment, exposure to unnecessary or harmful treatment, poorer quality of life, and loss of productivity [2, 6]. For healthcare systems, this failure can result in ineffective organisations and unnecessary expenditure [2, 6].

In healthcare, evidence-based practice refers to the translation or implementation of clinical research and knowledge into healthcare practice [6]. The two key steps toward evidence-based practice are: first, the translation of basic scientific knowledge to clinical practice, and secondly, the implementation of evidence-based practices that have found to be effective in the local setting into routine healthcare and policy [6, 11]. Barriers to successful implementation can be individual, structural, and organisational cultural [6, 12], including commitment from management, access to research, capacity issues, financial disincentives, inadequate skills within an organisation, or a lack of requisite facilities or equipment, staffing, peer morale and commitment, and leadership [6, 12]. Implementation strategies and frameworks assume or include important roles for leaders. Leadership has been shown to be an integral factor in nurturing a culture of evidence-based practice in clinical settings including cancer care, substance abuse, weight management, palliative care, and physiotherapy [3, 13–18]. Subsequently, leadership behaviours can encourage or discourage change and innovation within healthcare organisations [13, 19].

Despite leadership being considered a determining factor in implementing and sustaining evidence-based practices [1, 4, 20–24], the term remains an ambiguous concept in research [16]. Leadership has been conceptualised as a series of inherent personal traits, as learned behaviours, and as responses to particular situations or contexts [23]. Various types of leadership have been proposed including transformational leadership, transactional leadership, distributive leadership, charismatic leadership, heroic leadership, empowering leadership, engaging leadership, authentic leadership, collective leadership, servant leadership and passive or avoidant leadership [25–29]. A systematic review by Reichenpfader et al. [16] found that in 17 studies in the field of implementation science, the term was used imprecisely and inconsistently [16]. For the purpose of this paper, the authors will use Reichenpfader et al.’s [16] definition of leadership, being “a process of exerting intentional influence by one person over another person or group in order to achieve a certain outcome in a group or organization”. Likewise, the authors will consider leaders to be those people who are considered to exert influence on group or organisational outcomes, be they formal or informal leaders.

Formal leaders or positional leaders - managers or supervisors whose responsibilities include the oversight of staff, budgets, and operations - have the ability to procure and disperse funding and resources, and design and enforce implementation policies [19, 30]. Formal leaders have the responsibility to ensure that healthcare organisations support the implementation of evidence-based practice through adequate funding and resources, supportive plans, practices, and strategies, as well as providing a work environment conducive to implementation [19]. The Consolidated Framework for Implementation Research (CFIR) [31] considers formal leaders to be the people who project manage and coordinate implementation. In healthcare settings the implementation of practice change in health often requires leadership from multiple professional groups including nurses, physicians and allied health [32]. Powell et al. (2015) have suggested implementation strategies that leverage formal leaders including recruiting, designating, and training leaders for the change [33].

However, it is not only formal leaders who influence implementation. Change champions, who may be formal or informal leaders and are also referred to as opinion leaders, implementation leaders, facilitators, and change agents throughout the literature [34], also play a critical role in effective implementation [3, 19, 30]. Change champions are people within an organisation who are invested in implementing change, work hard to bring that change to fruition, are often personable, and are influential [3, 34]. Change champions may be frontline staff who may or may not have a formal management role, who frequently positively influence others’ attitudes or behaviours [3, 6, 30, 34]. Change champions acquire their influence through their demonstration of technical competence and accessibility and availability to their peers [6]. The CFIR suggests formal or informal change champions in implementation are those who are dedicated to supporting and driving implementation and influence attitudes toward implementation [31]. Implementation strategies utilising change champions identified by Powell et al. [33] include: identifying change champions, preparing them for the intervention and ensuring they are informed so they may influence the support of their colleagues [33]. It is these champions who have the responsibility to facilitate healthcare organisation climates being implementation-friendly through gaining support from senior management, formal leaders, as well as their peers [19].

Despite the critical role of both formal and informal leaders in facilitating the implementation of evidence-based practice in healthcare organisations, there is relatively little empirical study of how various aspects of leadership may be directly related to the efficacy or speed of research translation, or to the delivery of evidence-based practice [2]. Although it is clear that leadership is critical in the successful implementation and the sustainability of innovations [1, 35], it is unclear how the leadership traits and behaviours can be identified, measured, and developed [2, 3, 5, 19].

Consequently, the study of the relationship between leadership and research translation in healthcare requires accurate and relevant leadership scales. Leadership and change management is a growing area of scholarship [36–41], and some progress has been made on beginning to identify and synthesise scales which measure leadership traits and behaviours and to validate the psychometric properties of these scales [42–44]. Given the need for a variety of health professionals to be involved in the leadership of practice change; a leadership scale cannot be considered valid and reliable for administration with health professionals, until it is tested with a broad cross-section of such health professionals. However, a systematic review of general implementation scales (i.e. not leadership-specific) has highlighted a gap in the development and availability of validated scales which can be applied to the assessment of leadership traits and behaviours [45]. This gap inhibits the ability of implementation researchers and health professionals to identify evidence-based traits and behaviours which can facilitate identifying formal and informal leaders who may be integral in the promotion and delivery of evidence-based healthcare.

## Methods

The aim of this systematic review was to identify published leadership scales that have psychometric properties (reliability, validity or acceptability) which have been assessed with clinical health professionals.

This review follows the Preferred Reporting Items for Systematic Reviews and Meta-Analyses (PRISMA) (see Additional File 1) [46]. The synthesis methods of this review were guided by Clinton-McHarg et al.’s [45] 2016 work which examined the psychometric properties of scales developed in public healthcare and community settings [45]. This review was registered with PROSPERO (Registration Number CRD42019121544).

### Search strategy

**Fig. 1:**
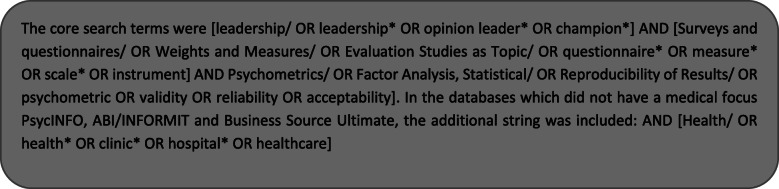
Search strategy

MEDLINE, EMBASE, PsycINFO, Cochrane, CINAHL, Scopus, ABI/INFORMIT, and Business Source Ultimate were searched to identify relevant studies published in English between January 2000 and December 2018. A second search was conducted with the same criteria between January 2019 and January 2020. These time periods were selected to optimise currency of the findings and given very few (if any) relevant studies were published prior to 2000. Prior to the database searches being conducted, search terms were developed through and iterative process guided by the PICO (problem, population, intervention and comparison, and outcome) Statement [47, 48]. These terms were refined in consultation with a senior librarian from the University of Newcastle, Australia to capture the relevant studies and to ensure the correct use of Boolean operators, truncation, and subject headings. The selected search terms for all databases related to the key concepts explored, being healthcare leadership (problem), health clinicians (population)_ the type of scale (intervention and comparison), and assessment of psychometric properties (outcome), with additional terms related to health included for non-health focussed databases (population). The full search strategy for the MEDLINE database is shown in Fig. 1.

### Eligibility

Publications were included if they: (1) were peer-reviewed journal articles reporting original research results; (2) reported data collected from or about practicing health professionals; (3) identified and assessed a leadership related scale for reliability, validity, or acceptability (See Table 1 for selection criteria and key definitions).

**Table 1:** Selection criteria key definitions

Key term	Definition
Peer-reviewed original literature	Peer-reviewed journal articles reporting original research results (i.e. the data or analysis is new).
Leadership scales	A scale was considered to be a leadership scale if the entire scale purported to assess leadership, or if one domain within the scale purported to assess leadership.
Psychometric properties	A scale was considered to have had its reliability, validity, or acceptability assessed if the study tested at least one of the following psychometric properties of a leadership scale or leadership domain: face-validity, content validity, construct validity, criterion validity, internal consistency correlations between a measure’s subscales and/or total scale, test-retest reliability, responsiveness, and/or acceptability.
Health professionals	Practicing health professionals including the following: doctor, nurse, midwife, psychologist, pharmacist, dietician/nutritionist, dentist, physiotherapist, radiation therapist, paramedic, occupational therapist, social worker, or disability worker.

### Study selection

**Fig. 2:**
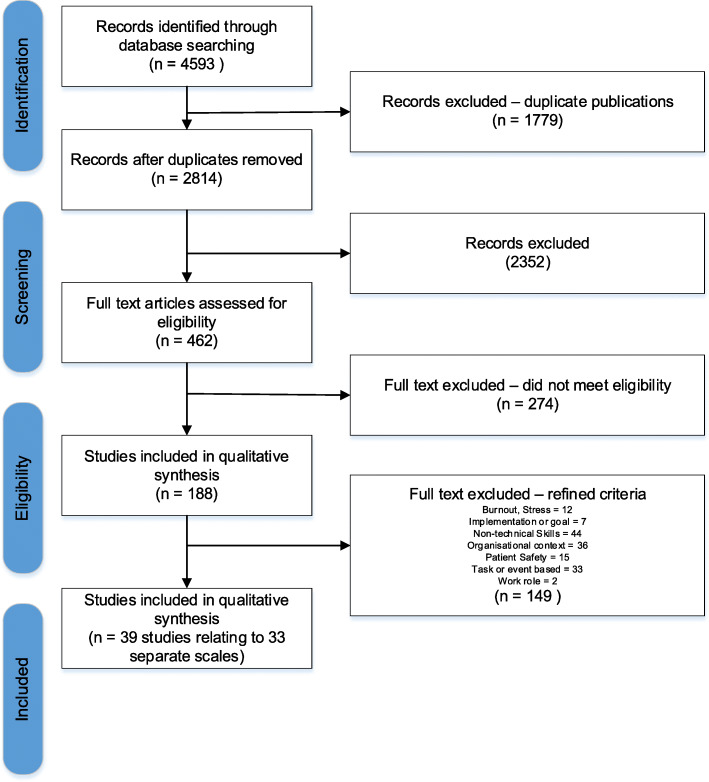
PRISMA flow diagram

The initial search yielded 4593 records. Of these, 1779 duplicate records were excluded. From the remaining pool of 2814 records, the titles and abstracts from a subset of 100 records that had been randomly selected were independently screened by two authors (CP and MC), to pilot the application of the inclusion and exclusion criteria. Title and abstracts from an additional subset of 500 randomly selected studies were then independently screened by the two authors (CP and MC) with the remaining screened by one author (MC). Studies that did not meet the inclusion criteria were excluded. The full-text manuscripts of the remaining 462 studies were then sourced. Of these 462 studies, the full text of 160 (~ 35%) studies were screened by two authors (MC and CP). The remaining 302 studies were screened by one author (MC). Of the 462 full text manuscripts screened, 274 did not meet the inclusion criteria and were subsequently excluded, leaving 188 eligible publications. After further discussion, the criteria for a leadership scale were refined to exclude any scales that did not specifically address leadership (i.e. those measuring burnout, implementation, non-technical skills, organisational context, patient safety, task/event-based leadership, or work roles). Using these criteria, a further 149 records were then excluded, leaving 39 records remaining for extraction (See Fig. 2 for PRISMA diagram).

### Data collection process & data items

The following information was extracted and tabulated from publications that met the inclusion criteria: (1) author(s); publication year; setting (e.g., oncology, cardiology, etc.); country of study; participants (e.g., physicians, nurses, multidisciplinary, etc.); study aim; methods; leadership assessment (namely, type and name of scale or tool); outcome assessment; and findings. And (2) psychometric properties including face validity, content validity, internal reliability, test-retest reliability, construct validity, criterion validity, responsiveness, acceptability, feasibility, revalidation, cross-cultural validation, convergent validity, and discriminant validity.

### Summary measures

#### Setting, sample, and characteristics of the innovation being assessed

Settings, sample, and characteristics of the innovation were extracted including the country and setting where the scale was validated, as well as the gender and profession of the sample and the sample response rate.

#### Face and content validity

Face validity assesses whether a scale is meaningful and relevant to those who use the scale [49]. Scales were considered to have face validity where administrators and/or test-takers agreed through a formal process that the scale measures what it is designed to measure [49]. Content validity assesses whether the scale fully captures the concept and sample it is designed to measure. The scale was considered to have content validity if the paper described how the items were selected and assessed, which revisions were made, and how they were made, or the theories and/or framework guiding the scale design [50].

#### Internal reliability and test-retest reliability

Scales or subscales were considered to have internal consistency if the Cronbach’s alpha was >.70 [51]. Where a paper only reported a range of Cronbach’s alphas for the scale’s subscales and part of the range was <.70, internal consistency was rejected. Repeated administration of a scale with the same sample and within 2–14 days was necessary to consider the scale’s test-retest reliability (i.e. a re-administration period outside of 2–14 days did not satisfy our criteria) [52]. Further, test-retest reliability was achieved if correlations between scores from the two administration time points had an intraclass correlation coefficient (ICC) of >.70 [45, 50].

#### Construct and criterion validity

Exploratory and/or confirmatory factor analysis (EFA/CFA) results were primarily used to determine a scale’s construct validity (i.e. internal structure). If both an EFA and CFA were conducted for a single scale, cut-offs were applied to the CFA results. When interpreting an EFA, scales were considered to have construct validity if eigenvalues were set at > 1 and/or > 50% of variance was explained by the scale [53, 54]. In studies where percentage of variance explained was reported, eigenvalues of > 1 were assumed. When interpreting a CFA, scales were considered to have construct validity where analysis was performed with a root mean square error of approximation (RMSEA) < .08 and a comparative fit index (CFI) > 0.95 [55, 56]. While a RMSEA of <.06 is supported by Clinton-McHarg (2016) [45], in this healthcare leadership literature, it was more common for an RMSEA of <.08 to be an acceptable cut-off, as often referenced from Hu and Bentler (1999) [56]. A scale was considered to have criterion validity if different scores were obtained for subpopulations with known differences (e.g., general nurse versus nurse manager) [57].

#### Responsiveness, acceptability, feasibility, revalidation, and cross-cultural adaptation

A scale’s ability to detect change over time (i.e. responsiveness) was determined based on a reported moderate effect size (> 5%) and/or minimal floor and/or ceiling effects (< 5%) [50, 58]. A scale was considered acceptable based on a low proportion of missing items and feasible based on time taken to complete, interpret, and score the scale. It was also noted if a scale was revalidated with additional populations or samples, or adapted across cultures or languages.

#### Convergent and discriminant validity

A scale’s convergent and discriminant validity was determined respectively by Pearson’s correlation coefficients (*r*) > .40 with similar scales and (r) < .30 with dissimilar scales. Where convergent or discriminant validity was reported for a scale, however testing did not involve correlating the scale with other similar/dissimilar validated scales, these were marked as unclear when determining satisfaction of criteria.

### Synthesis of results

Given that the publications varied considerably in their use and description of methodologies and measurements, a narrative synthesis rather than a meta-analysis was required. Popay et al. (2006:5) suggest that unlike a narrative review, which ‘are typically not systematic or transparent in their approach’, [59] narrative synthesis denotes ‘a process of synthesis that can be used in systematic reviews focusing on a wide range of questions, not only those relating to the effectiveness of a particular intervention … [It] is part of a larger review process that includes a systematic approach to searching for and quality appraising research-based evidence as well as the synthesis of this evidence’. [59] For the purpose of this review, studies were synthesised according to their expressed aim(s).

## Results

Of the 2814 records screened at the title and abstract stage, 2352 records were excluded. The 462 records remaining were screened at the full text stage. Of those records, 274 were excluded, leaving 188 eligible publications. After further discussion, the criteria for a leadership scale were refined to exclude any scales that did not specifically address leadership (i.e., those measuring burnout, implementation, non-technical skills, organisational context, patient safety, task/event-based leadership, or work roles). Using these criteria, a further 149 records were then excluded, leaving 39 unique records remaining for extraction (See Fig. 2 for PRISMA diagram).

### Study characteristics

#### Setting and characteristics of study sample for assessed scale

Of the 33 scales, the majority of scales were validated in English speaking countries including the USA (*n* = 15) and Canada (*n* = 4), but also with some translations and use in Europe and Asia. The Implementation Leadership Scale was validated with five separate types of health professionals, more than any other of the included 33 scales. This was followed by the Multifactorial Leadership Questionnaire and the Evidence-Based Practice Nursing Leadership Scale, which were both validated in two separate types of health professionals. The majority of studies validated scales with nurses (*n* = 27), followed by allied health (*n* = 10), with only two studies validating scales with a sample that included physicians; and no scales were validated with most other types of health professionals. It is also worth noting that women were overwhelmingly represented within the sample. The percentage of women in the studies ranged from 39% to 99.5%, with the average percentage of women across the 26 studies that reported gender being 75%. Given that the studies with the lowest rates of women in their samples were those studies that included non-nurse health professionals, this is likely due to nursing being a female-dominated profession. These data were reported in Table 2.

**Table 2:** Characteristics of study sample for assessed scales

Scale name	Measures	Scale details	Country	Sample size	Response rate	Gender of participants	Profession of participants
Aspiring leaders in Healthcare - Empowering individuals, Achieving excellence, Developing talents (AHEAD) [60]	Self-evaluated leadership competency in existing and emerging AHP leaders.	• 25 items • Likert (5-point) •2 Domains: Values (inc. 9 subdomains; e.g., Commitment; Compassion, Integrity) and Skills (inc. 16 subdomains; e.g., Creativity; Talent Management; Writing)	Singapore	106	Not reported	Females = 68%	Allied Health Professionals
Authentic Leadership Inventory (ALI) [61]	Employee’s perspectives on their managers’ authentic leadership characteristics.	• 14 items • Likert (5-point) • 5 Domains: Self-Awareness; Balanced Processing; Moral/Ethical Behaviour; and Relational Transparency	USA	*n* = 85	Not reported	Not reported	Nurses
Authentic Leadership Self-Assessment Questionnaire (ALSAQ-P) [62]	Self-evaluated authentic leadership.	• 16 items • Likert (5-point) • 4 Domains: Self-Awareness; Internalised Moral Perspective; Balanced Processing; Relational Transparency	Poland	*n = 3299*	Not Reported	Female = 98.5%	Registered Nurses
Authentic Nurse Leadership Questionnaire (ANLQ) [63]	Self-evaluated authentic leadership in nurse leaders.	• Item number not specified • response scale not specified • 5 Domains: Self-Awareness; Moral Ethical; Relational Integrity; Shared Decision Making; Caring	USA	*n* = 309	Not reported	Female = 94%	Registered Nurses
Charismatic Leadership Socialised Scale [64]	Charismatic leadership in nurse managers.	• 143 items • Likert (7-point) • Two parts: 1. Behaviour of the manager: Dimensions of the Charismatic and Instrumental Leadership 2. Behaviour of employees: Size of Commitment and Satisfaction, Motivation and Team Effectiveness	Brazil	*n* = 211	Not reported	Female = 81.5%	Registered Nurses
Clinical Leadership Needs Analysis (CLeeNA) [65]	Self-reported leadership needs of nurses and midwives.	•103 items •Likert (7-point) •7 Domains: Self and Team Development; Staff and Care Delivery; Technology and Care Initiatives; Financial and Service Management; Leadership and Clinical Practice; Patient Safety and Risk Management; and Standards of Care	Ireland	*n* = 321	~ 14.7%	Female = 91%	Registered Nurses
Clinical Leadership Survey (CLS) [66]	Self-evaluated clinical leader behaviours in staff nurses (basis in transformational leadership).	• 41 items • Likert (5-point) • 5 Domains: Challenging the Process; Inspiring a Shared Vision; Enabling Others to Act; Modelling the Way; and Encouraging the Heart	Canada	*n* = 480	41%	Female = 97%	Registered Nurses
Clinician Safety Culture and Leadership Questionnaire [67]	Clinician report of safety culture in hospitals at organisational and departmental levels, across teamwork, safety climate and leadership in healthcare.	• Clinician report • 35 items • Likert (5-point) • 3 Domains: Perceived Quality of Collaborating between Clinicians; Organisational Commitment to Patient Safety and Management Style; and Effectiveness of Healthcare Leaders in their Workplace	Australia	*n* = 1134	Not reported	Female = 39%	Physicians Nurses Allied Health Professionals
Cotter Preceptor Selection Instrument (CPSI) [68]	NPD practitioner perceptions of nurse attributes to assist nurse preceptor selection.	• 14 items • Likert (3-point) • 10 Domains: Clinical Competence; Nursing Process; Transformational Leadership; Collaboration/Communication Skills; Professional Development; Conflict Resolution; Commitment; Flexibility; Empowerment; and Values	USA	*n* = 13	Not reported	Not reported	Nurses
Director of Nursing Survey: Importance of Role Competencies [69]	DON’s Perceived importance of DON role competencies.	• 28 items • Likert (6-point) • 5 Domains: Transformational Leadership Forces; Structural Empowerment Forces; Exemplary Professional Practice Forces; New Knowledge, Innovation, and Improvements Force; and Empirical Quality Outcomes Force	China	*n* = 208	69%	Female = 99.5%	Directors of Nursing
Evidence-Based Practice Nursing Leadership Scale [70]	Staff nurse perceptions of support provided by unit level nurse managers to engage in EBP.	• 10 items • Response scale not specified • Item examples: ‘My manager provides time for me to engage in EBP’ and ‘My manager makes sure that I have access to relevant research on my unit’	USA	*n* = 422	24%	Not reported	Registered Nurses
Evidence-Based Practice Nursing Leadership Scale (Chinese adaptation) [71]	(As above)	(As above)	China	*n* = 419	96%	Female = 96%	Registered Nurses Senior Nurses Nurse Supervisors or above
Healthcare Evaluation & Assessment of Leadership (HEAL) [72]	Self-evaluated leadership competency based on the core principles of patient centeredness.	• 24 items • Likert (5-point) • 5 Domains: Critical Thinking; Emotional Intelligence; Teamwork; Selfless Service; and Integrity	USA	*n* = 126	63%	Female = 50%	Health professionals (internal medicine, paediatrics, surgery, radiology)
Human Capital Competencies Inventory (HCIC) [73]	Self-evaluated capital sustainability leadership attributes.	• 16 items • Likert (5-point) • 4 Domains: Ethical Leadership; Sustainable Leadership; Mindful Leadership; Servant Leadership	USA	*n* = 99	45%	Not reported	Nurse Managers
Human Capital Sustainability Leadership Scale (HCSLS) [74]	Self-evaluated capital sustainability leadership attributes.	• 16 items • Likert (5-point) • 4 Domains: Ethical Leadership; Sustainable Leadership; Mindful Leadership; Servant Leadership	Italy	EFA *n* = 207 CFA *n* = 274	Not reported	Female = 65%	Leaders from public and private health and care organisations
iLead [75]	Employees’ perspectives on their managers’ active and passive implementation specific leadership attributes.	• 20 items • Likert (5-point) • 2 Domains: Active Leadership; and Passive Leadership • Active Leadership Subdomains: Exemplary Behaviours; Individualised consideration; Intellectual Stimulation; and Contingent Reward • Passive Leadership Subdomains: Passive Management-by-Exception’ and Laissez-Faire	Sweden	*n* = 336	75% to study, of those 41% answered scale	Female = 90%	Health professionals (primary, psychiatric, rehabilitation, acute hospital care, and others)
Implementation Leadership Scale (ILS) [19]	Clinician reports of strategic leadership attributes in supervisors, specific to EBP implementation.	• 12 items • Likert (5-point) • Domains: Proactive Leadership; Knowledgeable Leadership; Supportive Leadership; and Perseverant Leadership	USA	*n* = 459	80%	Female = 79%	Mental Health Clinicians
Implementation Leadership Scale (ILS) [76]	(As above)	(As above)	USA	*n* = 323	Not reported	Female = 63%	Alcohol and Other Drug Treatment Professionals
Implementation Leadership Scale (ILS) [77]	(As above)	(As above)	USA	*n* = 214	93%	Female = 92%	Child welfare workers
Implementation Leadership Scale (ILS) [78]	(As above)	(As above)	USA	S1 *n* = 200 S2 *n* = 284	Not reported	Not reported	Registered Nurses
Implementation Leadership Scale (ILS) [79]	(As above)	(As above)	USA	*n* = 136	88%	Female = 76%	Mental health supervisors
Kuopio University Hospital Transformational Leadership Scale (KUHTLS) [80]	Nurse staff reports of manager’s/unit director’s transformational leadership attributes.	• 47 items • Likert (5-point) • 7 Domains: Decision; Appreciation; Growth; Justice; Performance; Individuality; and Administration	Cyprus	*n* = 315	79%	Female = 49%	Nurses
Leadership and Management Inventory (LaMI) [81]	Self-evaluation by superiors and/or subordinates of leadership skills and abilities of first-line nurse managers.	• 56 items • Likert (5-point) • 6 Domains: Organisation and Assignment; Information and Communication; Social Skills and Interpersonal Relations; Development and Support of Subordinates; Co-operation; and Analysis and Decisions	Sweden	S1 *n* = 149 S2 *n* = 197	S1 = 50% S2 = 87.5%	Not reported	Registered Nurses Healthcare Personnel from unspecified professions
Leadership Behaviour Description Questionnaire (Modified Version) [82]	Subordinate’s preference for specific behaviours from their leaders.	• 15 items • Likert (5-point) • Leadership behaviours and organisational commitment	India	*n* = 50	Not reported	Not reported	Paramedical Professionals
Leadership Competency Inventory (LCI) [83]	Self-reports from either individual staff members or managers on perceived degree of importance and development need for listed job competencies.	• 32 items • Likert (5-point) • 5 Domains: Personal Mastery; Managing Processes; Managing Resources; Leadership; and Managing Relationships	USA	*n* = 323	Not reported	Not reported	Nursing homes and hospitals
Leadership Influence over Professional Practice Environments Scale [84]	Leaders’ perceptions of their influence over the PPEs they oversee.	• 59 items • Likert (5-point) • 6 Domains: Collegial Administrative Approach; Internal Strategy and Resolve; Authority; Access to Resources; Leadership Expectations of Staff; and Status	USA	*n* = 150	Not reported	Female = 70%	Health Professionals (Vice president, chief nurse, associate chief nurse)
Leadership Practices Inventory (LPI) [43]	Original LPI recommended when used as an educational tool.	Original LPI • 30 items • Likert (5-point) • 5 Domains: Challenging the Process; Inspiring a Shared Vision; Enabling Others to Act; Modelling the Way; and Encouraging the Heart	Canada	*n* = 67	61%	Not reported	Nurses
	Newly derived LPI recommended for use in nursing research to measure leadership practices of established and aspiring nurses.	Newly Derived LPI • 27 items • Likert (10-point) • 3 Domains: Cognitive; Behavioural; Supportive					
Multifactor Leadership Questionnaire [85]	Both leader self-evaluation and subordinate evaluation of their supervisor’s transformational, active and passive leadership attributes.	• 78 items • Likert (5-point) • 9 Domains: Idealised Influence (Attributed); Idealised Influence (Behaviour); Inspiration Motivation; Intellectual Stimulation; Individualised consideration; Contingent Reward; Active Management-by-Exception; Passive Management-by-Exception; and Laissez-Faire Leadership	Canada	*n* = 378	38%	Female = 94%	Registered Nurses
Multifactor Leadership Questionnaire [86]	(As above)	(As above)	Finland	*n1* = 423 *n2* = 78	*n1* = 73%	Female = 95%	Nurses
Nurse Leadership and Organisational Culture (N-LOC) [87]	Self-reported attributes of leadership style and organisational culture.	• 62 items • Likert (7-point) • Domains: Leadership Style; and Organisational Culture	Hong Kong	*n1* = 295 *n2* = 1146	*n1* = 63% *n2* = 72%	*n1* Female = 89% *n2* Female = 88.5%	Nurses
Quantum Leadership Scale [88]	Nursing administrators’ quantum skills, leadership characteristics and functions.	• 37 items • Likert (5-point) • 3 Domains: Quantum Skills; Quantum Leaders’ Characteristics; and Quantum, Leaders’ Functions	Iran	*n* = 25	100%	Female & Married = 80%	Nurse administrators
Questionnaire on Self Perception of Nurses of Exercise of Leadership and (QUEPTAEEL) Questionnaire on Perception of Nurse Technicians and LPNs of Exercise of Leadership (QUAPEEL) [89]	Perception of leadership practice and coaching processes from the perspective of leaders (nurses; QUAPEEL) and followers (nurse technicians and LPNs; QUEPTAEEL).	Both Scales: • 20 items • Likert (6-point) and open- and closed-ended questions • Knowledge about leadership; and questions regarding abilities/attitudes of leaders/followers in coaching leadership practice	Brazil	*n* = 887	Not reported	Not reported	Nurses Nurse Technicians
Spiritual Leadership Questionnaire [90]	Nurses’ spiritual leadership attributes.	• 35 items • Likert (5-point) • 8 Domains: Vision; Faith and Hope; Altruism; Inner Life; Calling; Membership; Organisational Commitment; and Productivity	Iran	*n* = 400	91%	Female = 75.5%	Nurses
Supportive Leadership Behaviour Questionnaire [91]	Supportive leadership behaviours.	• 20 items • Likert (5-point) • 4 Domains: Support for Development; Integrity; Sincerity; and Recognition	Iran	*n* = 731	94%	Not reported	Head nurses Nurses
Supportive Supervisory Scale [92]	Supervisory support and ability to develop and maintain positive relationships.	• 15 items • Likert (5-point) • 2 Domains: Respects Uniqueness; and Being Reliable	Canada	*n* = 22	Not reported	Not reported	Healthcare Aides
Survey of Transformational Leadership [93]	Approaches to the conceptualization and measurement of transformational practices	• 84 items • Likert (5-point) • 5 Domains: Idealised Influence; Intellectual Stimulation; Inspirational Motivation; Individualised Consideration; and Empowerment	USA	*n = 214*	Staff = 62% Leaders = 57%	Female = 63.5%	Substance Use Workers
Sustainment Leadership Scale (SLS) [94]	Sustainment leadership attributes in first-level leaders and staff perceptions of leadership during sustainment.	• 12 items • Likert (5-point) • Four Domains: Proactive Leadership; Knowledgeable Leadership; Supportive Leadership; and Perseverant Leadership	USA	*n* = 157	95%	Not reported	Child Welfare Workers
Unnamed leadership questionnaire for dental practitioners [95]	Good and poor leadership.	• 61 items • Likert (7-point) • Domains: Challenges the notion of leadership domains and uses two constructs: Good Leadership and Poor Leadership	Turkey	*n* = 237	23%	Female = 40%	Dental Practitioners
Unnamed scale developed for study [96]	Organisational culture, leadership behaviours and job satisfaction.	• 60 items • Likert (5-point) • Domains: Organisational Culture; Leadership Behaviour; Job Satisfaction	Taiwan	*n* = 200	67%	Female = 99.5%	Nurses

Psychometric properties of the scales including face and content validity, internal reliability, test-retest reliability, construct and criterion validity, responsiveness, acceptability, feasibility, revalidation and cross-cultural validation, were assessed and reported in Table 3.

**Table 3:** Summary of psychometric properties reported for each scale

Title	Face validity	Content validity	Construct validity	Criterion validity	Internal consistency	Test-retest reliability	Responsiveness	Acceptability/ feasibility	Revalidation/ cross-cultural	Convergent	Discriminant
Aspiring leaders in Healthcare - Empowering individuals, Achieving excellence, Developing Talents (AHEAD) [60] (Allied Health Professionals)	–	Y	N*	U	Y	–	–	–	–	Y	–
Authentic Leadership Inventory (ALI) [61] (Nurses)	–	Y	Y	–	Y	–	–	–	–	–	–
Authentic Leadership Self-Assessment Questionnaire (Polish language version) (ALSAQ-P) [62] (Registered Nurses)	–	Y	Y	Y	Y	Y	Y	–	Y	–	–
Authentic Nurse Leadership Questionnaire (ANLQ) [63] (Registered Nurses)	–	Y	U	–	Y	–	–	–	–	Y	–
Charismatic Leadership Socialised Scale (adaptation for Brazilian culture) [64]	Y	Y	N	–	N	–	–	–	N	–	–
Clinical Leadership Needs Analysis Instrument (CLeeNA) [65] (Registered Nurses)	–	Y	Y	–	Y (only subscales reported)	–	–	–	–	–	–
Clinical Leadership Survey (CLS) [66] (Registered Nurses)	Y	Y	Y	–	N	–	–	–	–	Y	–
Clinician Safety Culture and Leadership Questionnaire [67] (Physicians, Nurses, Allied Health Professionals)	–	Y	N	–	Y (only subscales reported)	–	N	Y	–	U	U
Cotter Preceptor Selection Instrument (CPSI) [68] (Nurses)	Y	Y	–	Y	Y (only subscales reported)	–	–	–	–	Y	–
Director of Nursing Survey: Importance of Role Competencies [69] (Directors of Nursing)	Y	Y	–	–	Y (only subscales reported)	–	–	–	Y	–	–
Evidence-Based Practice Nursing Leadership Scale [70] (Registered Nurses)	Y	Y	Y	–	Y	–	–		–	–	–
The Evidence-Based Practice Nursing Leadership Scale (Chinese Adaptation) [71] (Registered Nurses, Senior Nurses, Nurse Supervisors or above)	Y	Y	Y	–	Y	Y	Y	–	Y	–	–
Healthcare Evaluation & Assessment of Leadership (HEAL) [72] (Health professionals (internal medicine, paediatrics, surgery, radiology))	–	Y	–	–	N	–	–	–	–	–	–
Human Capital Competencies Inventory (HCCI) [73] (Nurse Managers)	Y	Y	–	Y	Y	–	–	–	–	–	–
Human Capital Sustainability Leadership Scale (HCSLS) [74] (Leaders from public and private health and care organisations)	–	–	N*	–	Y (only subscales reported)	–	–	–	–	Y	–
iLead [75] (Health professionals (primary, psychiatric, rehabilitation, acute hospital care, and others)	Y	Y	N*	Y	Y	–	–	–	–	Y	Y
Implementation Leadership Scale (ILS) [76] (Alcohol and Other Drug Treatment Professionals)	–	–	N	–	Y (only subscales reported)	–	–	–	–	Y	U
Implementation Leadership Scale (ILS) [77] (Child Welfare Workers)	–	–	Y	–	Y (only subscales reported)	–	–	–	–	–	–
Implementation Leadership Scale (ILS) [78] (Registered Nurses)	Y	Y	Y	–	Y	–	–	–	–	–	–
Implementation Leadership Scale (ILS) [79] (Mental Health Supervisors)	–	–	N	–	Y (only subscales reported)	–	–	–	–	Y	Y
Implementation Leadership Scale (ILS) [19] (Mental Health Clinicians)	Y	Y	Y	–	Y	–	–	–	–	Y	Y
Kuopio University Hospital Transformational Leadership Scale (KUHTLS) [80] (Nurses)	–	–	N	–	Y	Y	–	–	Y	–	–
The Leadership and Management Inventory (LaMI) [81] (Registered Nurses, Healthcare Personnel)	–	Y	Y	–	Y (only subscales reported)	–	–	–	–	–	–
Leadership Behaviour Description Questionnaire (Modified Version) [82] (Paramedical Professionals)	–	–	U	–	Y (only subscales reported)	–	–	–	–	–	–
Leadership Competency Inventory (LCI) [83] (Nursing Homes and Hospitals)	Y	Y	Y	–	Y	–	–	–	–	–	–
The Leadership Influence Over Professional Practice Environments Scale [84] (Health professionals: Vice President, Chief Nurse, Associate Chief Nurse)	–	Y	Y	–	Y	–	–	–	–	–	–
Leadership Practices Inventory (LPI) (3-factor) [43] (Nurses)	–	Y	Y	–	Y	–	–	–	–	–	–
Multifactor Leadership Questionnaire (MLQ) [85] (Registered Nurses)	–	Y	Y	–	Y		–	–	–	–	–
Multifactor Leadership Questionnaire (MLQ) (6-factor) [86] (Nurses)	Y	Y	Y	–	Y (only subscales reported)	N	–	–	–	–	–
Nurse Leadership and Organisational Culture (N-LOC) Questionnaire (2-factor) [87] (Nurses)	–	Y	N*	–	Y	N	–	–	U	U	U
Quantum Leadership Scale [88] (Nurse Administrators)	Y	Y	–	–	–	Y	–	–	–	–	–
Questionnaire on Self-Perception of Nurses of Exercise of Leadership (QUAPEEL) [89]	Y	Y	–	–	Y	–	–	–	–	–	–
Spiritual Leadership Questionnaire (SLQ) [90] (Nurses)	Y	Y	N*	–	Y	Y	Y	Y	Y	–	–
Supportive Leadership Behaviours Questionnaire (SLB) [91] (Nurses, Head Nurses)	Y	Y	Y	–	Y	Y	–	–	Y	–	–
Supportive Supervisory Scale (SSS) [92] (Healthcare Aides)	Y	Y	U	–	Y	Y	–	–	–	–	–
Survey of Transformational Leadership (STL) [93] (Substance Use Workers)	Y	Y	Y	Y	Y (only subscales reported)	–	–	–	–	Y	–
Sustainment Leadership Scale (SLS) [94] (Child Welfare Workers)	–	Y	Y	–	Y	–	N	Y	–	–	–
Unnamed Leadership Questionnaire for (Dental Practitioners) [95]	–	Y	N	–	Y (only subscales reported)	–	–	–	–	–	–
Unnamed Scale Developed for the Study (Hospital Nurses) [96]	–	Y	Y	–	Y (only subscales reported)	–	–	–	–	–	–

#### Face and content validity

Of the 39 studies, face and content validity were evaluated and satisfied in 18 and 33 studies (16 and 30 scales), respectively.

#### Internal reliability

Of the included 33 scales, 29 scales (88%) achieved internal consistency, as indicated by Cronbach’s alphas >.70. All five studies reporting on the ILS indicated adequate internal consistency [19, 76–79], with two reporting for the entire scale [19, 78], and three for individual subscales (e.g. ‘Y (only subscales reported)’) [76, 77, 79]. Of the two studies reporting on the MLQ, one reported adequate internal consistency of the whole scale [85] and one of the individual subscales [86]. Of the remaining 27 scales that reported internal consistency, 16 reported for the entire scale [43, 60–64, 66, 70–73, 75, 78, 80, 83, 84, 87, 89], and ten for individual subscales [65, 67–69, 74, 81, 82, 93, 95, 96]. Three papers [64, 66, 72] reported only the range of Cronbach’s alpha values of the scale’s subscales, indicating one or more subscales with a Cronbach’s alpha of <.70, and thus did not satisfy our criteria for confirming the whole scale’s internal reliability.

#### Test-retest reliability

Of the 33 included scales, nine scales were tested for test-retest reliability [62, 71, 80, 86, 88, 90–92]. Considering the Pearson’s correlation coefficient cut-off of >.70 alone, seven scales achieved adequate test-retest reliability [62, 71, 80, 88, 90–92] and two did not [86, 87]. Re-administration periods ranged from within 2–14 days (*n* = 5) [71, 88, 90–92], between 14 and 30 days (*n* = 3) [62, 80, 87], and one year [86]. Our criteria for adequate test-retest reliability required both an *r* of >.70 and a re-administration period of between 2 and 14 days. The five scales re-tested within 2–14 days [71, 88, 90–92] fulfilled this criterion. One scale [80] demonstrated high test-retest reliability (*r* = .96) slightly outside the recommended re-administration period (15 days post-initial assessment), and was deemed successful in satisfying our criteria.

#### Construct and criterion validity

Thirty-three studies reported their scale’s internal structure using either an EFA (*n* = 10) [includes PCA [*n* = 7]]), a CFA (*n* = 10), or both (*n* = 12). Of the five studies [19, 76–79] reporting on the ILS, three [19, 77, 78] reported acceptable thresholds for good construct validity and two [76, 79] did not. Of the remaining 26 scales, 54% (*n* = 14) satisfied the acceptable thresholds for good construct validity, in that the EFA indicated > 50% of variance explained by the final model and eigenvalues were set at > 1 and/or the CFA indicated acceptable RMSEA (< .08) and CFI (> .95) values. Five scales were marked as marginally unsuccessful (i.e. ‘N*’) [60, 74, 75, 87, 90] in satisfying our criteria for construct validity, indicating either an RMSEA value <.08 but not <.06, and/or a CFI value >.90 but not >.95. One study [63] reported only the scale’s RMSEA value (< .08) and so, was marked as unclear (‘U’) when determining adequacy of construct validity (i.e. needing both the RMSEA and CFI to determine adequacy). Two further scales [82, 92] were marked ‘U’ as, although mentioning factor analysis or construct validity, they did not report RMSEA or CFI values. Four scales [64, 67, 80, 95] did not satisfy our criteria for adequate construct validity.

Of the 33 included scales, five scales [62, 68, 73, 75, 93] demonstrated criterion validity and one [60] was marked as unclear. Ten scales were correlated against existing scales to evaluate convergent and/or discriminant validity, as indicated by Pearson’s correlations (*r*). Eight of these scales (including the ILS, as convergent validity was tested and achieved in three of the five ILS studies) [60, 63, 66, 68, 74–76, 93] were considered to have convergent validity (*r* > .40) and two scales (the iLead and the ILS) [19, 75, 79] were considered to have both convergent and discriminant validity (*r* < .30). Three studies [67, 76, 87] reported on convergent and/or discriminant validity that did not involve correlating the scales with other validated scales and thus, were marked unclear (‘U’). Only one scale (Survey of Transformational Leadership) [93] achieved acceptable construct, criterion, and convergent validity.

#### Responsiveness, acceptability, feasibility, revalidation, and cross-cultural adaptation

Of the 39 studies, only five reported on responsiveness, three of which included scales that satisfied our criteria for floor and ceiling effects of < 5% [62, 71, 90]. One scale [67] had a small ceiling effect with scores skewed toward the higher end of the scale (14–62% of people obtaining the highest possible score for each item). The three papers that reported on their scale’s acceptability [67, 90, 94] satisfied low proportions of missing items. Only one study recorded the time taken to complete the scale (5–10 min) [67]. Other studies mentioned the expected time to complete the test in their methodology but did not record actual time taken by test-takers. Of the eight scales that underwent a process of revalidation in additional settings and subpopulations, five were successful in language retranslation and use with additional populations [62, 69, 71, 90, 91], two were unsuccessful within our criteria [64, 80] and one was unclear [87].

## Discussion

The objective of the review was to inform healthcare implementation regarding appropriate scales for assessing traits and behaviours for identifying formal or informal leaders who can successfully implement change. Notably, a large number of scales (*n* = 33) were identified as having undergone some form of psychometric testing with health professionals. However, only three of the scales had been tested on multiple occasions. These were the Implementation Leadership Scale (*n* = 5), the Multifactor Leadership Scale (*n* = 2), and the Evidence-Based Practice Nursing Leadership Scale (*n* = 2). The implementation Leadership Scale was found to have sound: face validity and content validity with Registered Nurses; construct validity with Child Welfare Workers, Registered Nurses, and Mental Health Clinicians; internal consistency with Child Welfare Workers, Registered Nurses, and Mental Health Clinicians; convergent validity with Mental Health Supervisors and Mental Health Clinicians. The Multifactor Leadership Questionnaire was found to have acceptable face validity, content validity, construct validity, and internal consistency with Nurses. The Evidence-Based Practice Nursing Leadership Scale was found to have acceptable face validity, content validity, construct validity, internal consistency, test-retestability, responsiveness, and was also cross-culturally validated. Most of the identified scales were tested in English speaking high-income countries such as the USA or Canada, predominantly with samples of nurses, or a sample of health professionals that included nurses (*n* = 27). Only two validation studies included physicians, which may suggest a limited number of scales proven suitable for assessing leadership in this group. Given that leadership roles can be occupied by physicians (e.g., department heads), nurses (e.g., nursing team leads) or others (e.g., rehabilitation team leads, mental health team leads) who are often involved in implementation of interventions, it is important that the scales for assessing leadership are tested in varied settings and known to be robust enough for research involving physicians, nurses, allied health professionals, and others who have a leadership role in practice change. It is also important to consider the roles of gender and cultural variation in leadership. Therefore, future work should consider validating leadership scales with a wider variety of diverse health professionals and in a variety of contexts.

The psychometric properties which were found to be strong for most scales, were content validity and internal consistency. These properties have similarly been found to be strong in the wider literature regarding testing of leadership scales with non-health professional samples [77, 97–100]. For example, the Servant Leadership Survey (SLS), which has been validated with 638 workers in three Spanish speaking countries (Spain, Argentina and Mexico) [99], the Ethical Leadership Behaviour Scale (ELBS) [98], which has been validated with 405 workers in Brazil, the School Counsellors Leadership Survey (SCLS) [97], which has been validated with 776 school counsellors and school counselling supervisors in the USA, and the Implementation Leadership Scale (ILS) [77], which has been cross-validated with 214 child-welfare providers in the USA. Glasgow et al. [101] suggest that a scale with acceptable internal consistency may also have a high number of items and consequently be more burdensome for users [101]. They further suggest it may be more pragmatic to consider content validity [101], which assesses how well the scale measures the concept and sample it is designed to measure. Content validity was strong in most (*n* = 30) scales in this study, including the Implementation Leadership Scale, Multi-Factor Leadership Questionnaire and Evidence-Based Practice Nursing Leadership Scale.

The findings in relation to construct validity are potentially concerning in that only 15 of the 33 scales were found to satisfy the acceptable thresholds for good construct validity. This potential concern has not been clearly identified in the literature regarding testing of leadership scales with non-health professional samples [102–104]. For example, one study found that although a more recent revision of the Multifactor Leadership Questionnaire (MLQ) exhibited high internal consistency, previous literature employed older versions that lacked discriminant validity [102]. Another study testing the construct validity of the Servant Leadership Scale (SLS) found the construct validity to be sound, however, the authors suggested that previous studies had not adequately tested the construct validity of the scale [71].

In relation to the remaining psychometric characteristics – test re-test reliability, responsiveness, acceptability, cross-cultural revalidation, convergent validity, discriminant validity and criterion validity – very limited testing has occurred.

There are seven scales that stand out as likely to be psychometrically sound for use with health professionals (at least for nurses and allied health professionals), in that they are reported to have satisfied most of the reliability and validity criteria. Of the scales tested in the English-language, the iLead scale demonstrated good internal reliability and face, content, criterion, convergent and discriminant validity, and was only marginally outside our cut-off for having satisfied construct validity (CFI > .90 but not >.95). It is important to note that several studies decided to deem a CFI of >.90 as adequate for good construct validity. The Supportive Leadership Behaviours Scale also satisfied internal and test-retest reliability, face, content, and construct validity, and was successfully revalidated. The Survey of Transformational Leadership (STL) demonstrated internal consistency and good construct, content, criterion, and convergent validity. Finally, the Implementation Leadership Scale has been evaluated several times and repeatedly demonstrates strong internal consistency, face and content validity, and convergent and discriminant validity. There are some inconsistencies in the scale’s construct validity, with two of the five evaluations of the ILS not satisfying our criteria for adequate construct validity. Of the scales tested in languages other than English, the Brazilian adaptation of the Charismatic Leadership Socialised Scale demonstrated inadequate construct validity and internal consistency, and so was not successfully revalidated. The Authentic Leadership Self-Assessment Questionnaire (Polish version) (ALSAQ-P) reported on and satisfied seven of the 11 criteria, including internal and test-retest reliability, content, construct and criterion validity, and evidence of good responsiveness and revalidation. The Persian version of the Spiritual Leadership Questionnaire (SLQ) demonstrated good internal and test-retest reliability and face and content validity. Moreover, the Persian SLQ was deemed responsive, acceptable and feasible, and achieved revalidation in Persian language. This scale, like the iLead scale, had a CFI of >.90 but did not meet our cut-off of a CFI > .95. The Chinese translation of the Evidence-Based Nursing Leadership Scale (EBP Nursing Leadership Scale) achieved internal and test-retest reliability, construct, face, and content validity, good responsiveness and revalidation. In summary, seven scales were found to have acceptable psychometric properties for use in healthcare, being the: Authentic Leadership Self-Assessment Questionnaire (Polish version), the iLead, the Spiritual Leadership Questionnaire (Persian version), the Supportive Leadership Behaviours Scale, the Evidence Based Nursing Scale (Chinese translation), and the Implementation Leadership Scale.

Few studies assessed the degree to which scale might be considered pragmatic, such as the time required to complete the scale or the acceptability and feasibility of the scale. Given the importance of identifying validated leadership scales in implementation science [45], and the key role of acceptability, feasibility, and cost (including time and resources) in assessing implementation outcomes [105], this represents a significant gap in the literature. However, it must be acknowledged that the search strategy did not focus extensively on pragmatic aspects of scales, for which tools are now emerging (e.g., Stanick, 2021) [106]. The availability of a quick, acceptable, and validated leadership scale would provide opportunities for researchers, leaders, and clinicians to assess health professionals in busy clinics for evidence-based leadership to drive evidence-based healthcare.

## Limitations

Due to the diversity of the literature on leadership, the chosen set of search terms may have excluded some relevant studies. The review inclusion criteria resulted in the exclusion of a large number of studies relating leadership in the context of developing or demonstrating specific or technical skills (e.g., surgical skills). While these types of scales were considered too narrow or purpose-specific to be of benefit for assessing healthcare leadership more generally, it is possible that these scales could be potentially useful if adapted or modified. In addition, as noted by a number of authors [101], the pragmatic aspects of scales are important for implementation but have not been thoroughly addressed here. Inclusion of such assessment would be a useful addition to the field. The assessment of construct validity in this review focussed on factor analysis, as this was the approach generally taken in these studies. It is acknowledged that other approaches such as assessing a construct’s relation to theory are also important to establishing construct validity.

Additionally, women were overwhelmingly represented in the samples, perhaps due to the high number of scales validated with nurses. A working paper by the World Health Organisation (WHO) analysed gender equity in health professionals in 104 countries [107]. They found that women make up 67% of health professionals in the included countries, however in most countries, occupations such as physicians, dentists and pharmacists are mostly dominated by men, with professions such as nursing and midwifery mostly comprised of women [107]. A 2017 systematic review of medical leadership in hospital settings [108] found 28 studies exploring physician leadership. Of those 28 studies, nine found ‘leading change’ to be described as an activity performed by physician leaders. This suggests there may be a role for physicians as formal or informal change champions. Boateng et al. [109] propose that one component of best practice of scale development and validation is to do so with the population it is intended to be used with. Given that most of these scales have been validated primarily with nurses and allied health professionals who are predominantly female, it is difficult to claim that these scales are suitable for assessing leadership traits and behaviours in healthcare professional groups which are mostly male, or professional groups other than nurses and allied health professionals. Therefore, future work may consider validating these scales with a wider variety of health professionals.

## Conclusion

There are seven scales which may be sufficiently sound to be used with nurses and allied health professionals. These are The Authentic Leadership Self-Assessment Questionnaire, the iLead scale, the Spiritual Leadership Questionnaire, the Supportive Leadership Behaviours Scale, The Survey of Transformational Leadership the Evidence-Based Nursing Leadership Scale and the Implementation Leadership Scale. There is a research gap in assessing leadership traits and behaviours of physicians and it appears that males have been underrepresented in some validation studies. Given the role of leadership in driving best practice in healthcare, there is a need for further psychometric assessment and validation of existing scales with physicians, males, and in assessing and understanding gender and cultural differences in implementation leadership. This serves to limit confidence with which the available scales can be used across health care disciplines in implementation research and practice, but also provides an opportunity for advancing the science of implementation leadership.

## Supplementary Information

**Additional file 1.** PRISMA 2009 Checklist.

## Data Availability

All data generated or analysed during this study are included in this published article and its supplementary information files.

## References

[1] Aarons GA, Farahnak LR, Ehrhart MG, Sklar M (2014). Aligning leadership across systems and organizations to develop strategic climate to for evidence-based practice implementation. Annu Rev Public Health.

[2] Gifford WA, Holyoke P, Squires JE, Angus D, Brosseau L, Egan M (2014). Managerial leadership for research use in nursing and allied health care professions: a narrative synthesis protocol. Syst Rev.

[3] Flodgren G, O'Brien MA, Parmelli E, Grimshaw JM. Local opinion leaders: effects on professional practice and healthcare outcomes. Cochrane Database Syst Rev. 2019;(6):CD000125. 10.1002/14651858.CD000125.pub5. 10.1002/14651858.CD000125.pub5 PMC6589938 31232458

[4] Gifford W, Davies B, Tourangeau A, Lefebre N (2011). Developing team leadership to facilitate guideline utilization: planning and evaluating a 3-month intervention strategy. J Nurs Manag.

[5] Gifford WA, Davies BL, Graham ID, Tourangeau A, Woodend AK, Lefebre N (2013). Developing leadership capacity for guideline use: a pilot cluster randomized control trial. Worldviews Evid-Based Nurs.

[6] Grimshaw JM, Eccles MP, Lavis JN, Hill SJ, Squires JE (2012). Knowledge translation of research findings. Implement Sci.

[7] McGlynn EA, Asch SM, Adams J, Keesey J, Hicks J, DeCristofaro A (2003). The quality of health care delivered to adults in the United States. N Engl J Med.

[8] McGlynn EA (2020). Measuring and improving quality in the US: where are we today?. J Am Board Fam Med.

[9] Schuster MA, McGlynn EA, Brook RH (2005). How good is the quality of health care in the United States?. Milbank Q.

[10] Runciman WB, Hunt TD, Hannaford NA, Hibbert PD, Westbrook JI, Coiera EW (2012). CareTrack: assessing the appropriateness of health care delivery in Australia. Med J Aust.

[11] Kitson A, Brook A, Harvey G, Jordan Z, Marshall R, O’Shea R (2018). Using complexity and network concepts to inform healthcare knowledge translation. Int J Health Policy Manag.

[12] Geerligs L, Rankin NM, Shepherd HL, Butow P (2018). Hospital-based interventions: a systematic review of staff-reported barriers and facilitators to implementation processes. Implement Sci.

[13] Choi M, Kim HS, Chung SK, Ahn MJ, Yoo JY, Park OS (2014). Evidence-based practice for pain management for cancer patients in an acute care setting. Int J Nurs Pract.

[14] Damschroder LJ, Hagedorn HJ (2011). A guiding framework and approach for implementation research in substance use disorders treatment. Psychol Addict Behav.

[15] Damschroder LJ, Lowery JC (2013). Evaluation of a large-scale weight management program using the consolidated framework for implementation research (CFIR). Implement Sci.

[16] Reichenpfader U, Carlfjord S, Nilsen P. Leadership in evidence-based practice: a systematic review. Leadersh Health Serv. 2015. 10.1108/LHS-08-2014-0061. 10.1108/LHS-08-2014-0061 26388219

[17] Nilsen P, Wallerstedt B, Behm L, Ahlström G (2018). Towards evidence-based palliative care in nursing homes in Sweden: a qualitative study informed by the organizational readiness to change theory. Implement Sci.

[18] Dannapfel P, Nilsen P (2016). Evidence-based physiotherapy culture: the influence of health care leaders in Sweden. Open J Leadersh.

[19] Aarons GA, Ehrhart MG, Farahnak LR (2014). The implementation leadership scale (ILS): development of a brief measure of unit level implementation leadership. Implement Sci.

[20] McGowan K (2016). Physical exercise and cancer-related fatigue in hospitalized patients: role of the clinical nurse leader in implementation of interventions. Clin J Oncol Nurs.

[21] Long JC, Cunningham FC, Wiley J, Carswell P, Braithwaite J (2013). Leadership in complex networks: the importance of network position and strategic action in a translational cancer research network. Implement Sci.

[22] Harvey G, Kitson A (2015). Translating evidence into healthcare policy and practice: single versus multi-faceted implementation strategies - is there a simple answer to a complex question?. Int J Health Policy Manag.

[23] Health Workforce Australia (2012). Leadership for the sustainability of the health system - part 1 - a literature review.

[24] Health Workforce Australia (2013). Health LEADS Australia: the Australian health leadership framework.

[25] Nawaz A, Khan I, ZA K (2016). Leadership theories and styles: a literature review. Leadership..

[26] Fischer SA (2016). Transformational leadership in nursing: a concept analysis. J Adv Nurs.

[27] Günzel-Jensen F, Jain AK, Kjeldsen AM (2016). Distributed leadership in health care: the role of formal leadership styles and organizational efficacy. Leadership..

[28] Luu TT, Rowley C, Dinh CK, Qian D, Le HQ (2019). Team creativity in public healthcare organizations: the roles of charismatic leadership, team job crafting, and collective public service motivation. Public Perform Manag Rev.

[29] Harris J, Mayo P (2018). Taking a case study approach to assessing alternative leadership models in health care. Br J Nurs.

[30] Gifford W, Lewis KB, Eldh AC, Fiset V, Abdul-Fatah T, Aberg AC (2019). Feasibility and usefulness of a leadership intervention to implement evidence-based falls prevention practices in residential care in Canada. Pilot Feasibility Stud.

[31] Damschroder LJ, Aron DC, Keith RE, Kirsh SR, Alexander JA, Lowery JC (2009). Fostering implementation of health services research findings into practice: a consolidated framework for advancing implementation science. Implement Sci.

[32] Daly J, Jackson D, Mannix J, Davidson PM, Hutchinson M (2014). The importance of clinical leadership in the hospital setting. J Healthc Leadersh.

[33] Powell BJ, Waltz TJ, Chinman MJ, Damschroder LJ, Smith JL, Matthieu MM (2015). A refined compilation of implementation strategies: results from the Expert Recommendations for Implementing Change (ERIC) project. Implement Sci.

[34] Miech EJ, Rattray NA, Flanagan ME, Damschroder L, Schmid AA, Damush TM (2018). Inside help: an integrative review of champions in healthcare-related implementation. SAGE Open Med.

[35] O'Reilly CA, Caldwell DF, Chatman JA, Lapiz M, Self W (2010). How leadership matters: the effects of leaders’ alignment on strategy implementation. Leadersh Q.

[36] van der Voet J (2014). The effectiveness and specificity of change management in a public organization: transformational leadership and a bureaucratic organizational structure. Eur Manag J.

[37] Karp T, Helgø TIT (2008). From change management to change leadership: embracing chaotic change in public service organizations. J Chang Manag.

[38] Kavanagh MH, Ashkanasy NM (2006). The impact of leadership and change management strategy on organizational culture and individual acceptance of change during a merger. Br J Manag.

[39] Gill R (2003). Change management - or change leadership?. J Chang Manag.

[40] Aarons GA, Ehrhart MG, Farahnak LR, Hurlburt MS (2015). Leadership and organizational change for implementation (LOCI): a randomized mixed method pilot study of a leadership and organization development intervention for evidence-based practice implementation. Implement Sci.

[41] Harden H, Fulop L (2015). The challenges of a relational leadership and the implications for efficacious decision-making in healthcare. Asia Pac J Health Manage.

[42] Barrett L, Plotnikoff RC, Raine K, Anderson D (2005). Development of measures of organizational leadership for health promotion. Health Educ Behav.

[43] Tourangeau AE, McGilton K (2004). Measuring leadership practices of nurses using the leadership practices inventory. Nurs Res.

[44] Carrara GLR, Bernardes A, Balsanelli AP, Camelo SHH, Gabriel CS, Anetti ACB (2017). Use of instruments to evaluate leadership in nursing and health services. Rev Gaucha Enferm.

[45] Clinton-McHarg T, Yoong SL, Tzelepis F, Regan T, Fielding A, Skelton E (2016). Psychometric properties of implementation measures for public health and community settings and mapping of constructs against the consolidated framework for implementation research: a systematic review. Implement Sci.

[46] Moher D, Liberati A, Tetzlaff J, Altman DG, PRISMA Group (2009). Preferred reporting items for systematic reviews and meta-analyses: the PRISMA statement. PLoS Med.

[47] Schardt C, Adams MB, Owens T, Keitz S, Fontelo P (2007). Utilization of the PICO framework to improve searching PubMed for clinical questions. BMC Med Inform Decis Mak.

[48] Huang X, Lin J, Demner-Fushman D (2006). Evaluation of PICO as a knowledge representation for clinical questions. AMIA Annu Symp Proc.

[49] Holden RR. Face validity. In: Winer IB, Craighead WE, editors. The Corsini Encyclopedia of Psychology [internet]. 4th ed. Wiley; 2010. p. 1–2. Available from:. 10.1002/9780470479216.corpsy0341.

[50] McDowell I (2006). Measuring health: a guide to rating scales and questionnaires.

[51] Taber KS (2018). The use of Cronbach’s alpha when developing and reporting research instruments in science education. Res Sci Educ.

[52] Marx RG, Menezes A, Horovitz L, Jones EC, Warren RF (2003). A comparison of two time intervals for test-retest reliability of health status instruments. J Clin Epidemiol.

[53] Costello AB, Osborne J (2005). Best practices in exploratory factor analysis: four recommendations for getting the most from your analysis. Pract Assess Res Eval.

[54] Beavers AS, Lounsbury JW, Richards JK, Huck SW (2013). Practical considerations for using exploratory factor analysis in educational research. Pract Assess Res Eval.

[55] Chen F, Curran PJ, Bollen KA, Kirby J, Paxton P (2008). An empirical evaluation of the use of fixed cutoff points in RMSEA test statistic in structural equation models. Sociol Methods Res.

[56] Hu L, Bentler PM (1999). Cutoff criteria for fit indexes in covariance structure analysis: conventional criteria versus new alternatives. Struct Equ Model Multidiscip J.

[57] Rubin A, Bellamy J (2012). Practitioner’s guide to using research for evidence-based practice.

[58] Husted JA, Cook RJ, Farewell VT, Gladman DD (2000). Methods for assessing responsiveness: a critical review and recommendations. J Clin Epidemiol.

[59] Popay J, Roberts H, Sowden A, Petticrew M, Arai L, Rodgers M (2006). Guidance on the conduct of narrative synthesis in systematic reviews: a product from the ESRC methods programme.

[60] Ang HG, Koh JM, Lee J, Pua YH (2016). Development and preliminary validation of a leadership competency instrument for existing and emerging allied health professional leaders. BMC Health Serv Res.

[61] Davidson ES, Mitchell A, Beverly C, Brown LM, Rettiganti M, Walden M (2018). Psychometric properties of the authentic leadership inventory in the nursing profession. J Nurs Meas.

[62] Panczyk M, Jaworski M, Iwanow L, Cieslak I, Gotlib J (2019). Psychometric properties of authentic leadership self-assessment questionnaire in a population-based sample of polish nurses. J Adv Nurs.

[63] Giordano-Mulligan M, Eckardt S (2019). Authentic nurse leadership conceptual framework: nurses’ perception of authentic nurse leader attributes. Nurs Adm Q.

[64] Ribeiro Chavaglia SR, Dela Coleta MF, Dela Coleta JA, Costa Mendes IA, Trevizan MA (2013). Adaptation and validation of the charismatic leadership socialized scale. Acta Paul Enferm.

[65] McCarthy VJ, Ashling M, Savage E, Hegarty J, Coffey, A, Leahy-Warren P, et al. Development and psychometric testing of the clinical leadership needs analysis (CLeeNA) instrument for nurses and midwives. J Nurs Manag. 2019;27(2):245–55. 10.1111/jonm.12672. 10.1111/jonm.12672 30171645

[66] Patrick A, Laschinger HK, Wong C, Finegan J (2011). Developing and testing a new measure of staff nurse clinical leadership: the clinical leadership survey. J Nurs Manag.

[67] Clay-Williams R, Taylor N, Ting HP, Winata T, Arnolda G, Braithwaite J (2020). The clinician safety culture and leadership questionnaire: refinement and validation in Australian public hospitals. Int J Qual Health Care.

[68] Cotter E, Eckardt P, Moylan L (2018). Instrument development and testing for selection of nursing preceptors. J..

[69] Spicer JG, Guo Y, Liu H, Hirsch J, Zhao H, Ma W (2010). Collaborative nursing leadership project in the People’s Republic of China. Int Nurs Rev.

[70] Pryse Y, McDaniel A, Schafer J (2014). Psychometric analysis of two new scales: the evidence-based practice nursing leadership and work environment scales. Worldviews Evid-Based Nurs.

[71] Zhang YP, Liu WH, Yan YT, Porr C, Zhang Y, Wei HH. Psychometric testing of the evidence-based practice nursing leadership scale and the work environment scale after cross-cultural adaptation in mainland China. Eval Health Prof. 2018:163278718801439. 10.1177/0163278718801439. 10.1177/0163278718801439 30301376

[72] Murphy KR, McManigle JE, Benjamin M, Wildman T, Jones AL, Dekker TJ (2016). Design, implementation, and demographic differences of HEAL: a self-report health care leadership instrument. J Healthc Leadersh.

[73] Donaher K, Russell G, Scoble KB, Chen J (2007). The human capital competencies inventory for developing nurse managers. J Contin Educ Nurs.

[74] Di Fabio A, Peiró JM. Human capital sustainability leadership to promote sustainable development and healthy organizations: a new scale. Sustainability (Switzerland). 2018;10(7). 10.3390/su10072413.

[75] Mosson R, von Thiele SU, Hasson H, Lundmark R, Richter A (2018). How do iLead? Validation of a scale measuring active and passive implementation leadership in Swedish healthcare. BMJ Open.

[76] Aarons GA, Ehrhart MG, Torres EM, Finn NK, Roesch SC (2016). Validation of the implementation leadership scale (ILS) in substance use disorder treatment organizations. J Subst Abus Treat.

[77] Finn NK, Torres EM, Ehrhart MG, Roesch SC, Aarons GA (2016). Cross-validation of the implementation leadership scale (ILS) in child welfare service organizations. Child Maltreat.

[78] Shuman CJ, Ehrhart MG, Torres EM, Veliz P, Kath LM, VanAntwerp K (2020). EBP implementation leadership of frontline nurse managers: validation of the implementation leadership scale in acute care. Worldviews Evid-Based Nurs.

[79] Torres EM, Ehrhart MG, Beidas RS, Farahnak LR, Finn NK, Aarons GA (2018). Validation of the implementation leadership scale (ILS) with supervisors’ self-ratings. Community Ment Health J.

[80] Sapountzi-Krepia D, Prezerakos P, Zyga S, Petrou A, Krommydas G, Malliarou M (2019). Psychometric properties of the Greek version of the Kuopio University Hospital Transformational Leadership Scale (KUHTLS). Int J Caring Sci.

[81] Skytt B, Carlsson M, Ljunggren B, Engstrom M (2008). Psychometric testing of the leadership and management inventory: a tool to measure the skills and abilities of first-line nurse managers. J Nurs Manag.

[82] Acharya R, Dasbiswas AK (2017). A study on the relationship between organizational commitment and leadership style on paramedical personnel in Kolkata. Int J Bus Insights Transformation.

[83] Joon Yoon H, Hoon Song J, Donahue WE, Woodley KK. Leadership competency inventory: a systematic process of developing and validating a leadership competency scale. J Leadersh Stud. 2010;4(3):39–50. 10.1002/jls.20176.

[84] Adams JM, Nikolaev N, Erickson JI, Ditomassi M, Jones DA (2013). Identification of the psychometric properties of the leadership influence over professional practice environments scale. J Nurs Adm.

[85] Boamah SA, Tremplay P. Examining the factor structure of the MLQ transactional and transformational leadership dimensions in nursing context. West J Nurs Res. 2018:193945918778833. 10.1177/0193945918778833. 10.1177/0193945918778833 29808783

[86] Kanste O, Miettunen J, Kyngas H (2007). Psychometric properties of the multifactor leadership questionnaire among nurses. J Adv Nurs.

[87] Lui JNM, Johnston JM (2019). Validation of the nurse leadership and organizational culture (N-LOC) questionnaire. BMC Health Serv Res.

[88] Dargahi H (2013). Quantum leadership: the implication for Iranian nursing leaders. Acta Med Iran.

[89] Cardoso ML, Ramos LH, D'Innocenzo M (2014). Coaching leadership: leaders’ and followers’ perception assessment questionnaires in nursing. Einstein..

[90] Zagheri Tafreshi M, Jahandar P, Rassouli M, Atashzadeh-Shoorideh F, Kavousi A. Psychometric properties of the Persian version of spiritual leadership questionnaire (SLQ): a methodological study. Iran Red Crescent Med J. 2017;19 (7)(no pagination):e55930. 10.5812/ircmj.55930.

[91] Shirazi M, Emami AH, Mirmoosavi SJ, Alavinia SM, Zamanian H, Fathollahbeigi F (2014). Contextualization and standardization of the supportive leadership behavior questionnaire based on socio- cognitive theory in Iran. Med J Islam Repub Iran.

[92] McGilton KS (2010). Development and psychometric testing of the supportive supervisory scale. J Nurs Scholarsh.

[93] Edwards JR, Knight DK, Broome KM, Flynn PM (2010). The development and validation of a transformational leadership survey for substance use treatment programs. Subst Use Misuse.

[94] Ehrhart MG, Torres EM, Green AE, Trott EM, Willging CE, Moullin JC (2018). Leading for the long haul: a mixed-method evaluation of the sustainment leadership scale (SLS). Implementation Sci.

[95] Hill H, Brocklehurst P (2015). Leadership in dentistry: findings from new tool to measure clinical leadership. J Healthc Leadersh.

[96] Tsai Y (2011). Relationship between organizational culture, leadership behavior and job satisfaction. BMC Health Serv Res.

[97] Young A, Bryan J (2015). The school counselor leadership survey: instrument development and exploratory factor analysis. Prof Sch Couns.

[98] Silva Filho ALA, Ferreira MC, Valentini F (2019). Validity evidence of the ethical leadership behavior scale (ELBS). Psico-USF..

[99] Rodríguez-Carvajal R, de Rivas S, Herrero M, Moreno-Jiménez B, Van Dierendonck D. Leading people positively: cross-cultural validation of the servant leadership survey (SLS). Span J Psychol. 2014;17. 10.1017/sjp.2014.73. 10.1017/sjp.2014.73 26055345

[100] Carmeli A, Reiter-Palmon R, Ziv E (2010). Inclusive leadership and employee involvement in creative tasks in the workplace: the mediating role of psychological safety. Creat Res J.

[101] Glasgow RE, Riley WT (2013). Pragmatic measures: what they are and why we need them. Am J Prev Med.

[102] Antonakis J, Avolio BJ, Sivasubramaniam N (2003). Context and leadership: an examination of the nine-factor full-range leadership theory using the multifactor leadership questionnaire. Leadersh Q.

[103] van Beveren P, Dimas ID, Lourenço PR, Rebelo T (2017). Psychometric properties of the Portuguese version of the global transformational leadership (GTL) scale. Rev Psicol Trab Organ.

[104] Preacher KJ, Zhang Z, Zyphur MJ (2016). Multilevel structural equation models for assessing moderation within and across levels of analysis. Psychol Methods.

[105] Proctor E, Silmere H, Raghavan R, Hovmand P, Aarons G, Bunger A (2011). Outcomes for implementation research: conceptual distinctions, measurement challenges, and research agenda. Adm Policy Ment Health Ment Health Serv Res.

[106] Stanick CF, Halko HM, Nolen EA, Powell BJ, Dorsey CN, Mettert KD (2021). Pragmatic measures for implementation research: development of the psychometric and pragmatic evidence rating scale. Transl Behav Med.

[107] Boniol M, McIsaac M, Xu L, Wuliji T, Diallo K, Campbell J (2019). Gender equity in the health workforce: analysis of 104 countries.

[108] Berghout MA, Fabbricotti IN, Buljac-Samardžić M, Hilders CG (2017). Medical leaders or masters?—a systematic review of medical leadership in hospital settings. PLoS One.

[109] Boateng GO, Neilands TB, Frongillo EA, Melgar-Quiñonez HR, Young SL (2018). Best practices for developing and validating scales for health, social, and behavioral research: a primer. Front Public Health.

